# Immunohistological assessment of the synovial tissue in small joints in rheumatoid arthritis: validation of a minimally invasive ultrasound-guided synovial biopsy procedure

**DOI:** 10.1186/ar2302

**Published:** 2007-09-28

**Authors:** Carlo Alberto Scirè, Oscar Epis, Veronica Codullo, Frances Humby, Patrizia Morbini, Antonio Manzo, Roberto Caporali, Costantino Pitzalis, Carlomaurizio Montecucco

**Affiliations:** 1 Chair and Division of Rheumatology, University of Pavia, Fondazione IRCCS Policlinico San Matteo, Piazzale Golgi 12, I 27100 Pavia, Italy; 2 Centre for Experimental Medicine and Rheumatology, 2^nd ^floor John Vane Science Centre, William Harvey Research Institute, St Bartholomew's and Royal London School of Medicine. Charter House Square, London, EC1M6BQ, UK; 3 Department of Pathology, University of Pavia, Fondazione IRCCS Policlinico San Matteo, Piazzale Golgi 12, I 27100 Pavia, Italy

## Abstract

The aim of the present study was to perform an immunohistological assessment of the synovial tissue from involved small joints in rheumatoid arthritis (RA) and to explore the reliability of a mini-invasive ultrasound (US)-guided technique of small joint synovial biopsy for the histopathological assessment. Synovial tissue collected during arthrotomic surgery of small joints in nine patients served as the gold standard for the validation of the histological assessment. Small hand-joint synovial biopsies from an additional nine patients with erosive RA were obtained by a mini-invasive US-guided procedure, performed percutaneously by the portal and rigid forceps technique. Using digital image analysis, the area fractions of synovial macrophages (CD68 cells), T cells (CD3 cells) and B cells (CD20 cells) were measured in all high-power fields of every sample at different cutting levels. The representative sample was defined as the minimal number of high-power fields whose mean area fraction would reflect the overall mean area fraction within a percentage mean difference of 10%. For each patient, a range of three to five large samples for surgical biopsies and a range of 8–12 samples for US-guided biopsies were collected and analysed. In arthrotomic samples, the analysis of a randomly selected tissue area of 2.5 mm^2 ^was representative of the overall value for CD68, CD3 and CD20 cells. US-guided samples allowed histological evaluation in 100% of cases, with a mean valid area of 18.56 mm^2 ^(range 7.29–38.28 mm^2^). The analysis of a cumulative area of 2.5 mm^2 ^from eight randomly selected sections (from different samples or from different cutting levels) allowed to reduce the percentage mean difference to less than 10% for CD68, CD3 and CD20 cells. In conclusion, US-guided synovial biopsy represents a reliable tool for the assessment of the histopathological features of RA patients with a mini-invasive approach.

## Introduction

A number of approaches to the assessment of the synovial membrane have been proposed in an attempt to establish the degree of inflammation and the phenotypic characterization of infiltrating cell subsets [1,2]. Of the cell types found in the synovium, the intensity of CD68-positive macrophage infiltration at baseline has been associated with progressive joint damage [3,4], and has been confirmed as an optimal biomarker of clinical response in several randomized clinical trials of both disease-modifying antirheumatic drugs and biologic agents [5-8].

The importance of the evaluation of the synovial membrane, particularly for clinical trials, has been reinforced by work demonstrating that changes in the synovial membrane are more reliable than clinical assessments, such as the disease activity score, when determining response to treatment [9]. In addition, the development of techniques such as digital image analysis has made the rapid reliable assessment of large areas of tissue a realistic proposition [10,11].

There are several possible approaches to the acquisition of synovial tissue, but arthroscopic biopsy is generally accepted as the gold standard [12], allowing for good quality, sizeable biopsy specimens. The knee joint has been the favourite biopsy site owing to the ease of arthroscopic access and to the knowledge that it appears representative microscopically of other synovial joints [13]. Several validation studies demonstrated the reliability of a multiple sampling of synovial membrane for immunohistological studies, inferring that synovial sampling from clinically involved knee joints might provide a picture of the disease for each patient at every disease phase [1,12,14-19].

The knee joint, however, is only involved in a subset of patients with early arthritis, and knee involvement at onset would appear to identify a cohort of patients with a more aggressive disease course [20]. Studies based exclusively on knee biopsy, at least in early arthritis, would therefore be inherently biased towards recruitment of patients with a worse prognosis. In addition, as the small joints of the hands and feet are most commonly involved in early arthritis and since associated outcome measures of erosive burden are assessed here, acquisition of the synovial membrane from these joints would appear imperative for high-quality translational research.

For these reasons, different biopsy techniques have been developed to acquire synovial tissue from small joints, both by needle and by arthroscopic approach [21,22]. The recent development of ultrasound (US)-guided synovial biopsy may help to overcome the blindness of the needle biopsy and the invasiveness of arthroscopic biopsy of small joints [23].

US-guided synovial biopsy of small joints is not, however, presently a standard technique in clinical practice – predominantly because of several still unanswered important issues regarding the pathologic variability in small joints and the validity of the technique to produce meaningful biological specimens.

The present study aimed to address a number of these issues: whether the analysis of randomly selected synovial tissue collected from small joints in rheumatoid arthritis (RA) patients could be representative of the overall inflammatory status of the joint; whether adequate synovial tissue could be obtained by US-guided synovial biopsy of small joints with regard to a series of standard immune (CD3 cells, CD20 cells, CD68 cells) and histological parameters; and to determine the minimum number of synovial biopsies under US guidance required to achieve reliable measurements of the above immune histological features.

For this purpose, we first analysed RA patient synovial membranes collected from surgical procedures to assess the minimum area of synovial tissue representative of the overall joint status for a series of standardized immunohistological parameters. We next tested the availability of that area in our synovial samples collected by the novel US-guided procedure, and evaluated in US-guided biopsies the variability of the main immune-phenotypic features to assess the number of samples needed to achieve reliable measurements.

## Materials and methods

### Patients

To assess the immunohistological features of rheumatoid synovitis, surgical synovial tissue specimens were obtained during arthroplasty or synovectomy from clinically involved (swollen) small joints of nine patients with longstanding erosive RA who fulfilled the 1987 American Rheumatism Association classification criteria [24].

To validate US-guided synovial biopsy, the procedure was performed in an additional nine patients with longstanding erosive RA before starting biologic agent treatment.

All patients gave their informed consent for biopsy, and the study was approved by the Local Ethical Committee. The main demographic and clinical characteristics of the patients as well as the biopsy sites are presented in Table 1.

**Table 1:** Clinicopathological characteristics of synovial tissues obtained by surgical biopsy and ultrasound-guided biopsy

Biopsy	Age (years)	Sex	Disease duration (years)	RF/aCCP	Disease-modifying antirheumatic drugs	Joint	Valid samples/total samples	Valid sections	Cumulative area (mm^2^)
S-SY1	65	Female	18	+/+	MTX	Wrist	3/3	9	24.47
S-SY2	44	Female	12	-/-	PDN	I MTP	3/3	9	37.72
S-SY3	63	Female	14	+/+	MTX + HCQ	II and III MTPs	3/3	9	44.02
S-SY4	46	Female	34	-/+	HCQ	Wrist	4/4	12	41.68
S-SY5	46	Female	17	-/-	LFN + PDN	Wrist	5/5	15	60.32
S-SY6	75	Female	7	-/-	MTX + PDN	MCPs	3/3	9	34.75
S-SY7	78	Female	8	-/-	MTX + PDN	MCPs	3/3	9	38.68
S-SY8	46	Male	2	-/+	MTX + PDN	MCPs	3/3	9	22.42
S-SY9	68	Female	5	-/-	MTX + HCQ + PDN	I MTP	3/3	9	45.01
U-SY1	23	Female	5	+/+	CYA + PDN	II MCP	4/8	12	16.86
U-SY2	59	Female	4	-/-	MTX + PDN	V MCP	5/8	15	17.64
U-SY3	64	Female	18	+/+	PDN	II PIP	10/12	30	38.28
U-SY4	61	Female	11	-/+	MTX + PDN	V MCP	3/12	9	7.29
U-SY5	69	Male	10	+/+	MTX + HCQ + PDN	II MCP	8/12	24	20.16
U-SY6	75	Male	11	+/+	HCQ + SLZ + PDN	V MCP	10/12	29	28.71
U-SY7	67	Female	7	-/+	MTX + HCQ + PDN	II MCP	8/12	23	8.82
U-SY8	62	Female	8	-/-	MTX + PDN	II MCP	7/12	20	8.43
U-SY9	61	Female	11	+/+	MTX + HCQ + PDN	II MCP	9/12	26	20.91

The good quality/overall number of collected sample rate and the number of valid sections obtained considering three different cutting levels are provided. The cumulative area is computed on overall sections for each patient. S-SY, surgical biopsy synovial tissue; U-SY, ultrasound-guided biopsy synovial tissue; RF/aCCP, rheumatoid factor/anticyclic citrullinated peptide antibodies; MTX, methotrexate: PDN, prednisone; HCQ, hydroxychloroquine; LFN, leflunomide; CYA, cyclosporine A; SLZ, sulfasalazine; MCP, metacarpophalangeal; MTP, metatarsophalangeal; PIP, proximal interphalangeal.

### Ultrasound-guided synovial biopsies

US-guided synovial biopsy was performed using the portal and forceps technique [23]. Briefly, US examination was performed with the Toshiba Nemio (Toshiba America Medical Systems, Inc. Tustin, CA, USA) using a multifrequency linear transducer (Hockey Stick Linear 8–14 MHz; Toshiba America Medical Systems). The transducer was used for standard Doppler-sonographic evaluation of the joint and for direct assistance of the biopsy procedure. Effusion and synovitis were identified and distinguished according to the following definition: effusion was defined as hypoechoic or anechoic compressible intra-articular material, within synovial recesses. Synovitis was defined as echogenic noncompressible intra-articular tissue, within synovial recesses. Power-Doppler variables were adjusted to the lowest permissible pulse repetition frequency to maximize the sensitivity. Low wall filters were used. The colour gain was set just below the level at which colour noise appeared underlying bone (no flow should be visualized at the bony surface). [25]

**Figure 1:**
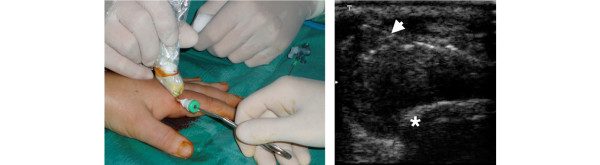
**Ultrasound-guided synovial biopsy of a second metacarpophalangeal joint using the portal and forceps technique**. Arrow, open forceps inside the joint; asterisk, metacarpal hand.

Under sterile conditions, US dorsal longitudinal guidance was used for metacarpophalangeal and proximal interphalangeal biopsies. The skin and subcutaneous tissue and the synovial space were infiltrated with 1–3 ml local anaesthetic (Xilonest 1%; Astrazeneca, Wedel, Germany) using a 25-gauge needle. After 2 minutes a 14-gauge needle was inserted into the planned area under direct US vision. A 6 F percutaneous sheath introducer (Cordis Corporation, Miami, FL, USA) was inserted into the joint under US guidance following a flexible wire. The flexible wire was then removed, and a rigid Hartmann's ear forceps (Medicon, Tuttlingen, Germany) was used for the biopsy procedures through the portal. Several independent samples (at least eight) could be taken through the same portal (Figure 1) from the hypertrophic synovium including power-Doppler-positive areas. The whole procedure was completed within 30 minutes and it was generally well tolerated. No procedure-related adverse events were recorded in our series.

### Synovial samples

Biopsy specimens were immediately fixed in 4% formalin for up to 24 hours. After paraffin embedding, 20 serial sections 5 μm thick (100 μm interval) from each sample at three different cutting levels were mounted onto glass slides.

With regard to the synovial surgical biopsies, slides obtained from all available specimens were analysed at three different cutting levels. Given the higher homogeneity of the cellular reaction in the upper subsynovial layer and the sampling potential of the US-guided biopsy, the proximal 600 μm of synovial sublining immediately adjacent to the lining layer was considered for the purpose of histomorphometric analysis [26].

Tissue sections from US-guided biopsy were considered valid for histological analysis only if an intact lining was visible on H & E-stained sections. All sections were coded and systematically examined throughout their entire area at three different cutting levels by a single, blinded observer (CAS).

### Immunohistochemistry

Formalin-fixed, paraffin-embedded tissue sections were deparaffinized and rehydrated through graded ethanol solutions, and were immunostained as previously reported [27]. Briefly, once hydrated the sections were heated for 35 minutes at 96°C in DAKO Target Retrieval Solution (S1699; DAKO, Carpinteria, CA, USA). The sections were then washed in Tris-buffered saline (pH 7.6) and incubated for 10 minutes with Protein Block Serum Free (X0909; DAKO). The following primary antibodies were used (2 hours of incubation): rabbit anti-human CD3 polyclonal antibody (immunoglobulin, A0452; DAKO), mouse anti-human CD20 antibody (IgG2a, clone L26; DAKO), and mouse anti-human CD68 antibody (IgG3, clone PG-M1; DAKO). Sections were then incubated with the appropriate biotinylated secondary antibody (E0431 swine anti-rabbit immunoglobulin, or Z0259 rabbit anti-mouse immunoglobulin; DAKO) for 30 minutes, followed by streptavidin biotin–alkaline phosphatase complex (K0391; DAKO) for an additional 30 minutes. Reactions were then developed using the New Fuchsin Substrate Kit (K0698; DAKO) and sections were counterstained with Meyer's Haematoxylin 0.1% (MHS-16; Sigma Diagnostics, St Louis, MO, USA) and mounted with Aquamount mounting medium (BDH, Poole, UK). Primary and secondary antibodies were diluted in DAKO Antibody Diluent (S3022; DAKO).

### Microscopic analysis

All high-power (× 40) microscopic fields (HPFs) were examined on an Olympus microscope (BX51; Olympus, Tokyo, Japan), captured using a digital camera (Olympus) and transferred to a computer platform. The resultant colour images were of dimension 2,048 × 1,536 pixels, RGB format, with a 24-bit-per-pixel resolution. For each acquisition session, the microscope, camera and computer were calibrated according to a standardized procedure. The images obtained were stored in an uncompressed TIFF format and were examined using the image-analysis system ImageJ 1.35 s (National Institutes of Health, Bethesda, MD, USA). Image segmentation was performed by RGB colour discrimination using threshold ranges such that a binary overlay was created covering only the positively stained areas. This threshold was determined by two distinct observers and was kept constant for all measurements for the same marker. A separate binary mask was created that identified the total tissue area in each image, so the final parameter of analysis was the area fraction. To speed up the image analysis process, all procedures were performed using an *ad hoc *macro program for each marker.

### Statistical analysis

The area fraction of immunoreactivity for each marker was measured on multiple HPFs for each patient. The interfield variability was determined as the percentage difference between the mean area fraction when all HPFs from each patient were considered and the mean area fraction calculated from randomly selected individual HPFs. For surgical biopsies, this analysis was performed with multiple sets of data (10 sets) of an increasing number of randomly chosen HPFs from all available specimens of each patient.

For US-guided biopsies the same analysis was performed with an increasing number of samples at different cutting levels. To increase the sampling efficiency, the number of HPFs required to reduce the percentage mean difference to less than 10% of the total sample mean was used as the variability threshold for the assay, as previously reported [26].

The variance of the measurements was reduced into its contributing factors (patient, sample and cutting level) and was analysed by analysis of variance using a general linear model and nested design. All samples at three different cutting levels were analysed for the computations. Both samples and cutting levels were nested in the patient variable. The *F *values for each analysis were provided. Differences were considered significant at *P *< 0.05. This variance component analysis was carried out using the STATISTICA data analysis software system (version 7.1, 2005; StatSoft, Inc., Tulsa, OK, USA).

## Results

### Determination of maximum sampling efficiency on surgical synovial samples

**Figure 2:**
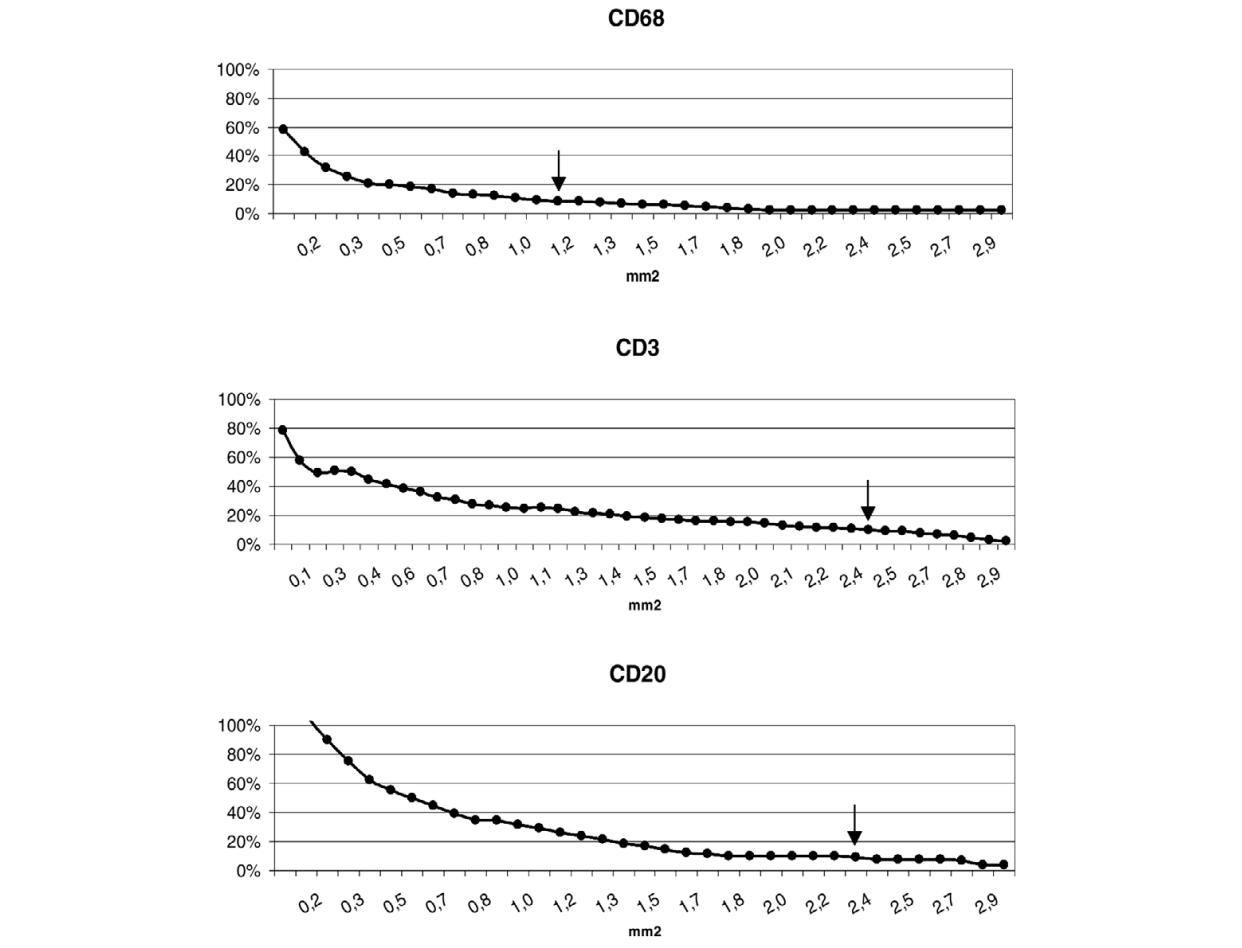
**Evaluation of the minimum area required for quantitative analyses of CD68, CD3 and CD20 cells**. For each of the tissue markers studied, a plot was generated to show the effects of increasing sample size (that is, the number of high-power fields examined) and the proximity of this sample mean from the overall determination of area fraction. Data represent the mean values of all cases. As the number of high-power fields examined increases, the difference between the sampling mean and the overall mean reduces. A threshold value of 10% of the overall mean (arrows) was set as providing a reasonable estimator of the true sample mean.

Figure 2 shows the effects of an increasing sample (HPF) number on the estimate of the overall sample mean for each immunostain. As the number of fields increases (and hence the fraction of the total sampling population used to create the area fraction calculation increases), the proximity of the sample mean to the overall mean area fraction is asymptotic. The number of HPFs required to reduce the sample mean to within 10% of the overall sample mean was set as the efficiency threshold for the analysis. Any further sampling analysis after this point would lead to an insignificant gain in the estimate of the mean area fraction for the particular tissue marker being examined.

It can be seen that the analysis of a cumulative area of 2.5 mm^2 ^(randomly selected from all available samples) is sufficient to reduce the variability of the estimate to within 10% of the total sample mean for all markers. Given the more homogeneous distribution of CD68-positive cells within the sublining, a variation of less than 10% was typically obtained by evaluation of only 1.2 mm^2^, while CD3 and CD20 cells needed a larger area because of their focal distribution in the sublining layer. These results provide essential baseline data for the comparative evaluation of different biopsy regimes and methodologies, and they represent a 'gold standard' for the development of a morphometric protocol for the biopsy of synovial tissue from the minor joints.

### Efficacy of ultrasound-guided small joint biopsies

**Figure 3:**
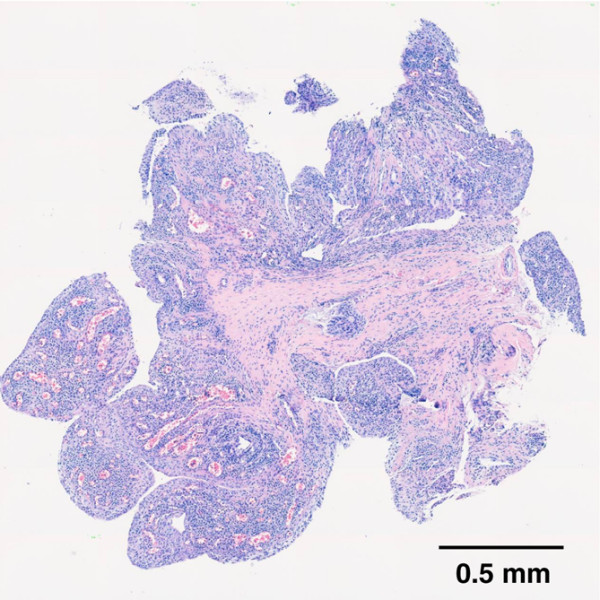
**Microphotograph of an ultrasound-guided sample**. H & E staining of a metacarpophalangeal sample of a rheumatoid arthritis patient (patient U-SY1), a result of multiple high-power fields (40× objective) merged into a single image (montage).

Qualitative histological examination of H & E-stained specimens showed that good quality synovial tissue was available in all cases of US-guided biopsy. A representative synovial sample obtained by US-guided biopsy from a small joint of a RA patient is illustrated in Figure 3. The histological validity and the amount of valuable synovial tissue are detailed in Table 1.

The success rate of the synovial sampling (that is, valid samples/total collected samples) ranged from 25% to 83%, with a mean value of 63% (95% confidence interval, 52–75%). The mean valuable area for each valid sample at a single cutting level was 0.88 mm^2 ^(95% confidence interval, 0.80–0.97 mm^2^), ranging from 0.16 to 3.19 mm^2^. By analysing three different cutting levels for each valid sample, the minimum useful area (2.5 mm^2^) for quantitative analysis was achieved in all patients (Table 1).

### Quantitative analysis in ultrasound-guided samples

The overall number of sections for each patient ranged from 9 to 30 (three cutting levels for each valid sample).

We first analysed the variability of each immunohistological parameter between patients, and between samples and cutting levels. Table 2 summarizes the results of the components of variance analysis. The observed differences were mainly due to interpatient differences, and secondarily to differences between samples or cutting levels in a similar way.

**Table 2:** Components of variance for each marker

Marker	Patients	Samples	Cutting levels
CD68 cells	34.44, *P *< 0.001	10.29, *P *< 0.001	13.00, *P *< 0.001
CD3 cells	23.22, *P *< 0.001	2.58, *P *< 0.001	5.51, *P *< 0.001
CD20 cells	16.11, *P *< 0.001	4.05, *P *< 0.001	3.77, *P *< 0.001

*F *value and significance level reported for each marker in each component of variance.

To estimate the number of sections to analyse in each patient to minimize intrapatient variability, we calculated the difference between the mean values in all sections and those obtained from a 2.5 mm^2 ^randomly selected area from an increasing number of sections.

**Figure 4:**
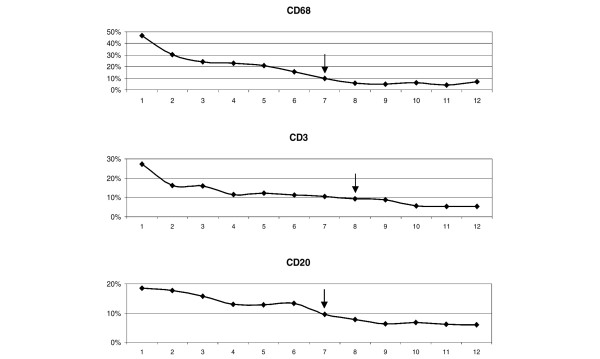
**Number of ultrasound-guided biopsy sections required for quantitative analysis**. Evaluation of the number of ultrasound-guided biopsy sections required for quantitative analyses of CD68, CD3 and CD20 cells. Reduction in the percentage mean difference can be obtained by studying 2.5 mm^2 ^from an increasing number of sections. Arrows, number of sections that allow one to achieve a percentage mean difference lower than 10%. *x *axis, number of sections studied; *y *axis, percentage mean difference.

The outcome of such analysis is depicted in Figure 4. From this figure it can be concluded that the percentage mean difference for the staining of a marker decreases below ± 10% when a minimum of eight samples are considered in the evaluation.

## Discussion

The results presented here show that the analysis of a small amount of synovial tissue is also representative of the joint status in small joints of RA patients, show that US-guided synovial biopsy at this site represents a reliable approach for good quality tissue collection, and show that quantitative immunohistological studies are feasible through the examination of multiple specimens obtained by US-guided biopsy of small joints.

Several studies have addressed the heterogeneity of cellular and molecular marker expression in RA synovial biopsies, investigating the amount of tissue or the number of samples needed to obtain reliable, reproducible results capable of detecting small changes within the synovial membrane [15-19,26]. One of the first studies that attempted to address this question was performed on synovial tissue from RA patients undergoing joint surgery [26], using a semiquantitative approach to estimate the degree of cellularity. The study demonstrated that the analysis of a cumulative area of 2.5 mm^2 ^from at least three biopsy specimens gave an accurate estimate of the overall joint. Analogous results have been reported on the synovium obtained from the knee joint either arthroplastically, arthroscopically or using blind needle biopsies, when considering T-cell infiltration, lining layer thickness or vascularity scores [15]. Our analysis focusing on synovial tissue obtained surgically from small joints reproduces these findings, demonstrating that the analysis of a 2.5 mm^2 ^tissue section allows an estimate of the number of macrophages (CD68-positive cells), T cells (CD3-positive cells) and B cells (CD20-positive cells) within 10% of the mean for the overall tissue. Although we limited our study to the examination of only these three cell subsets, it is widely recognized that the combination of CD68 cells, CD3 cells and CD20 cells, which are highly variable focal parameters, gives a biologically relevant assessment of the overall cellular infiltration within the synovium.

Macrophage infiltration is currently regarded as the main histopathological marker of activity and severity in RA [6,28,29]. In addition, despite the rapidly growing interest in the pathogenic role of B cells within the synovial membrane [30], the present study is the first to specifically address the question of what constitutes a representative synovial sample analysis for B-cell infiltration.

There have been numerous previous studies attempting to standardize the quantity of synovial tissue required to achieve a representative measure of the overall joint, the majority using an arthroscopic approach to obtain synovial tissue from knee joints and hence able to determine the number of biopsies from exact sites within the joint to allow accurate histopathologcial evaluation of the synovial membrane [12]. The recent development of a novel minimally invasive technique of US-guided synovial biopsy has been reported in the literature [23,31], and includes assessment of the small joint biopsy with success rates in acquisition of histologically reliable tissue ranging from 89% to 93%. Among 120 US-guided biopsies, however, only one report is made of metacarpophalangeal and metatarsophalangeal joint biopsy. Our study therefore describes the largest case series of small joint synovial US-guided biopsies in RA. The collection of several independent samples (up to 12 samples) is feasible and allows a high histological success rate (100% in our series). Since the procedure is minimally invasive, repeated biopsies could be planned to monitor the disease course and/or the response to therapy.

A basic stereological rule for the analysis of all tissue states that the degree of variation is greatest between individuals and is least between sections from the same biopsy. This stereological rule was elegantly demonstrated for synovial tissue by Dolhain and colleagues, who looked at the degree of T-cell infiltration within and between multiple biopsy sites [14]. We used a similar approach to address the problem of variability in cellular infiltrates in biopsies obtained under US guidance from small joints, and we came to similar conclusions demonstrating that the main component of the variance was due to the differences between patients rather than between samples or cutting levels of the same sample. We concluded that eight different sections obtained from either different samples or different cutting levels are required to reduce the sampling error to less than 10% for a reliable analysis of CD68, CD3 and CD20 cells. In our series, considering one cutting level in five out of nine cases, two cutting levels in eight out of nine cases, and three cutting levels in all cases produced a reliable result.

The limitation of the methodology used in this study mainly results from the analysis of a heterogeneous group of RA patients and from the application of results derived from surgical biopsies (from a different set of patients) to US-guided biopsies. Performing US-guided biopsy and surgery on the same patient, however, can be easily appreciated as far from simple, from both a practical point of view and from an ethical point of view. In addition, the benefit of our approach is that we provide data applicable to synovial samples from patients with different disease durations and different pharmacological treatments, which maximizes differences between patients [1], thus increasing the representativeness of our study. Moreover, the bias in the evaluations of joint replacement synovial tissue is limited in our series because, as in US-guided biopsies, synoviectomy or arthroplasty in small joints were performed in active diseases, differing from large joint surgery where it is generally performed in end-stage disease [3].

## Conclusion

In summary, the present study shows that US-guided biopsy of synovial hand joints in RA patients is a reliable tool for histological evaluation. If 12 different samples are taken, a valid assessment at least for CD20 cells, CD3 cells and CD68 cells is possible.

These findings are comparable with those obtained when synovial tissue from the knee is examined, and are the first attempt to standardize the minimum requirements for analysis of the small joints of the hands. The study of the synovial tissue from small joints can be a valuable research tool, allowing for this tissue to be incorporated into future trial designs, which is critical for further understanding of the pathogenesis of this disease and for assessing marker changes in the course of disease or in response to targeted therapies.

## Abbreviations

H & E = haematoxylin and eosin; HPF = high-power field; RA = rheumatoid arthritis; US = ultrasound.

## Competing interests

The authors declare that they have no competing interests.

## Authors' contributions

CAS substantially contributed to the conception of the study, and the acquisition, analysis and interpretation of data. OE substantially contributed to acquisition of tissue specimens. VC contributed to the acquisition and interpretation of the results. FH participated in drafting the manuscript. PM substantially participated in the methodological aspect of the study. AM substantially contributed to interpretation of the data. RC contributed to interpretation of the data and to critical review of the manuscript. CP substantially contributed to the critical review of the manuscript. CM provided final approval of the version to be published.
